# GM-CSF Signalling Boosts Dramatically IL-1Production

**DOI:** 10.1371/journal.pone.0023025

**Published:** 2011-07-28

**Authors:** Hanif Javanmard Khameneh, Siti Aminah Bte Mohammad Isa, Lin Min, Fam Wee Nih, Christiane Ruedl

**Affiliations:** Nanyang Technological University, School of Biological Sciences, Singapore; Singapore Institute for Clinical Sciences, Singapore

## Abstract

GM-CSF is mostly known for its capacity to promote bone marrow progenitor differentiation, to mobilize and mature myeloid cells as well as to enhance host immune responses. However the molecular actions of GM-CSF are still poorly characterized. Here we describe a new surprising facet of this “old” growth factor as a key regulator involved in IL-1βsecretion. We found that IL-1β release, a pivotal component of the triggered innate system, is heavily dependent on the signaling induced by GM-CSF in such an extent that in its absence IL-1β is only weakly secreted. GM-CSF synergizes with LPS for IL-1β secretion mainly at the level of pro-IL-1β production via strengthening the NF-κB signaling. In addition, we show that expression of Rab39a, a GTPase required for caspase-1 dependent IL-1β secretion is greatly augmented by LPS and GM-CSF co-stimulation suggesting a potential GM-CSF contribution in enhancing IL-1β exocytosis. The role of GM-CSF in regulating IL-1β secretion is extended also in vivo, since GM-CSF R−/− mice are more resistant to LPS-mediated septic shock. These results identify GM-CSF as a key regulator of IL-1β production and indicate GM-CSF as a previously underestimated target for therapeutic intervention.

## Introduction

Dendritic cells (DCs) and macrophages (MØ), the major sentinels of the innate immune system, are probing and recognising microbes and danger signals via specific pattern-recognition receptors. The resultant intracellular signalling cascades lead to the maturation and to secretion of several pro-inflammatory cytokines, such as IL–1 (IL-1α and β), TNF-α, IL-6. All these cytokines are ultimately involved in the pathogen elimination by promoting and sustaining an effective adaptive immune response [1].

IL-1β is a particularly potent pyrogenic cytokine and a key modulator involved in the regulation of immune responses against a variety of microbes as well as in several acute and chronic inflammatory disorders and septic shock [2]. Differently to other pro-inflammatory cytokines, the secretion of the active form of IL-1 is a tightly controlled two-step process which includes (i) the synthesis of the pro- IL-1β and (ii) its processing into a mature active IL-1β followed by its secretion. The generation of the active IL-1β via cleavage of its cytoplasmic proform is strictly dependent on caspase-1, a component of multi protein complexes, the best characterized being the NLRP3 inflammasome [3] [4].

The tightly controlled production and secretion of IL-1β typically requires two separate “external” signals. The first signal, provided by several pathogen-associated molecular patterns (PAMPs), promotes transcription, production and intracellular accumulation of the cytokine pro-form. The second signal, sometimes called “danger” signal is mediated by ATP, uric acid and aluminium salt (Alum) and many other factors, is needed for NLRP3 and caspase-1 activation and subsequent release of the biological active form of the cytokine. To avoid uncontrolled NLRP3 priming, its activation is strictly dependent on the PAMP-mediated NF-κB activation which regulates also the expression of NLRP3 [5], [6].

This multi-level control ensures that the highly pyrogenic and inflammatory IL-1β is secreted only under circumstances when an inflammatory response is required. The loss of this stringent control can lead to autoinflammatory disorders, such as familial Mediterranean fever, familial cold autoinflammatory syndrome and Muckle-Wells syndrome. All these inflammatory diseases respond to specific IL-1β or caspase-1 rather than TNF-α blocking therapies [7]. Hence a better understanding of the pathways controlling inflammasome activation and IL-1β release will potentially lead to the identification of new targets for therapeutic intervention.

Although GM-CSF was originally characterized as a haematopoietic growth factor responsible for the differentiation of bone marrow (BM) progenitor cells [8], in the last years it has been recognized as a key pro-inflammatory cytokine during inflammation or in response to infection [9]. A variety of studies have shown its contribution to promoting survival and activation of MØ, neutrophils, eosinophils and DCs, in mobilizing myeloid populations into the blood [10] as well as in enhancing host defense mechanisms against various bacterial and fungal infections [11]. In this study, we have identified a new function for GM-CSF as a crucial factor in the regulation of the multi-step process required for an efficient IL-1 release. Antigen presenting cells exploit GM-CSF as a licensing factor, which controls the transcriptional induction of IL-1β. This surprising new insights into “an old factor” high-lights the recently emerging picture of GM-CSF as a front-line cytokine driving inflammation.

## Results

### GM-CSF boosts dramatically LPS-induced IL-1 secretion

**Figure 1:**
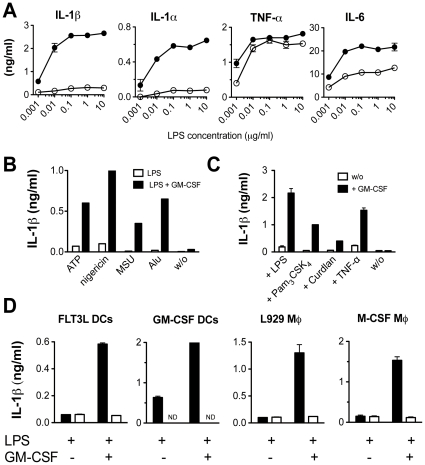
GM-CSF boosts LPS-induced IL-1 secretion. (A) CD11b^+^ fraction of FLT3L derived DCs was stimulated 24 h with a wide range of different LPS concentrations (0.001–10 µg/ml) in absence (white circles) or presence of 5 ng/ml GM-CSF (black circles). 5 mM ATP was added as a danger signal. Released IL-1β, IL-1α, TNF-α and IL-6 were measured in the culture supernatants by standard ELISA and each value represents the mean of triplicates +/− SD. (B) CD11b^+^ fraction of FLT3L generated DCs was primed for 24 h with 100 ng/ml LPS with (back bars) or without (white bars) 5 ng/ml GM-CSF and stimulated with different danger signals (5 mM ATP, 1 µM nigericin, 100 µg/ml MSU, 200 µg/ml Alu). Each bar represents the mean of triplicates +/− SD. (C) CD11b^+^ fraction of FLT3L generated DCs was primed with TLR agonists (100 ng/ml LPS and Pam_3_CSK_4_), Dectin agonist, Curdlan (100 µg/ml) and pro-inflammatory cytokine TNF-α (100 ng/ml) in absence (white bars) or presence (back bars) of 5 ng/ml GM-CSF and stimulated subsequently with ATP. Each bar represents the mean of triplicates +/− SD. (D) GM-CSF derived BM DCs, M-CSF-derived BM MØ as well as L929-derived BM MØ were compared to the CD11b^+^ fraction of FLT3L-derived DCs for their capacity to secrete IL-1β upon 24 h LPS stimulation (100 ng/ml) in absence or in presence of GM-CSF (5 ng/ml). ATP was added as danger signal. Both, WT (black bars) and GM-CSF R−/− cells (white bars) were tested. Each bar represents the mean of triplicates +/− SD. All results are representative of at least two independent experiments.

**Figure 2:**
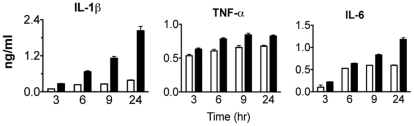
CD11b^+^ fraction of FLT3L derived DCs were stimulated for 3, 6, 9 and 24 h respectively with LPS alone (white bars) or in combination with GM-CSF (black bars). For the detection of IL-1β, 4×10^5^ cells were stimulated for 1 h with ATP, for IL-6 and TNF-α the cytokine release of 1×10^5^ cells was analysed without ATP treatment. Released cytokines were measured by ELISA. Each bar represents the mean of triplicates +/− SD.

To test the effects of GM-CSF on the cytokine production in “danger situation” we stimulated FLTL3- derived DC in the presence of ATP with LPS alone or in combination with GM-CSF. Cultures were then measured for their contents of several pro-inflammatory cytokines, such as of IL-1α, IL-1β, TNF-α, and IL-6. GM-CSF alone did not induce any cytokine release but to our surprise combination with LPS dramatically enhanced the secretion of IL-1s with all tested concentrations of LPS about ten fold (Fig. 1A). Interestingly, no effect was seen for TNF-α release, while IL-6 production was only slightly increased (2-fold) (Fig. 1A). GM-CSF did not act as a danger signal itself since it always needs to partner with ATP or any other tested known danger signals, such as nigericin, MSU or Alum (Fig. 1B), whereas, as expected , TNF-α and IL-6 were released (Fig. 2, middle and right panel). Furthermore, we observed that the synergistic effect of GM-CSF was not restricted to classical TLR-agonists, such as LPS, Pam3CysSK4, since GM-CSF strongly amplified also the IL-1β secretion in response to other non TLR-specific microbial stimuli such as the Dectin-1 ligand Curdlan as well as TNF-α cytokine (Fig. 1C).

We then extended our analysis to other APCs, such as GM-CSF derived BM DCs, M-CSF-derived BM MØ as well as L929 conditioned supernatant -derived BM MØ, focusing on the IL-1β secretion profile. In this study, we did not analyse the secretion profile of neutrophils, known to be one of the major IL-1β producers, since they secrete Il-1β independently from caspase-1 [12] which is indispensable for MØ and DCs. In all cases striking synergism as above was observed (Fig. 1D). As expected the same target cell populations derived from GM-CSFR−/− BM cells failed to respond robustly (Fig. 1D). Interestingly, strongest responses were generated by GM-CSF derived DCs which were probably optimally primed already during their generation. Of note, robust (ng/ml level) IL-1 responses observed in earlier reports can probably be attributed to residual presence of GM-CSF. In absence of this growth factor, IL-1 levels tend to reach much lower levels in the pg/ml range.

To exclude the acquisition of tolerance due to prolonged stimulation with LPS [13], we tested shorter incubation periods ranging from 3 h to 9 h. Similarly to the results obtained after 24 h LPS treatment, higher levels of IL-1β were detectable only when FLT3L-derived CD11b^+^ DCs were co-incubated with GM-CSF (Fig. 2, left panel). In the case of the other measured cytokines, TNF-α and IL-6, the effect of GM-CSF on their release was moderate and comparable the result described in Fig. 1A (Fig. 2, middle and right panel).

Interestingly GM-CSF proved to be the most potent modulator of IL-1β secretion, since other known inflammatory cytokines did not mediate the same effect in DCs. In fact, none of the tested cytokines including M-CSF, IL-6, IFN-γ and TNF-α stimulated efficiently DCs to release high amounts of IL-1β when co-applied with LPS (Fig. S1).

Taken together, these results indicate that require GM-CSF for a robust IL-1β secretion in response to several microbial and non-microbial stimuli.

### GM-CSF synergises LPS-induced pro-IL-1β synthesis via increased activation of NF-κB but does not affect caspase-1 cleavage

An efficient IL-1β secretion requires aNFκB-dependent synthesis of pro-IL-1β followed by a required danger signal which mediates the activation of the caspase-1.

**Figure 3:**
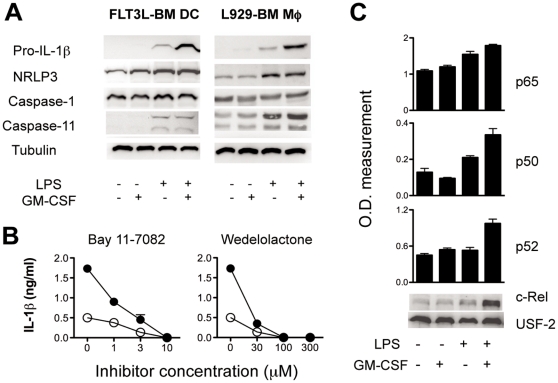
GM-CSF amplifies LPS-induced pro-IL-1β synthesis via enforcement of NF-κB activation. (A) Western blot analysis of pro-IL-1β, NLRP3, caspase-1 and -11 in FLT3L derived CD11b^+^ DCs and L929-derived BM MØ. Cells were left untreated or stimulated overnight with 100 ng/ml LPS, 5 ng/ml GM-CSF or a combination of both and cell lysates were subsequently prepared and separated on a 10% SDS-PAGE gel. (B) Dose-dependent inhibition of IL-1β secretion via two different NF-κB inhibitors, Bay 11-7082 and Wedelolactone. FLT3L derived DCs were pre-treated for 30 min with different concentration range of inhibitors, stimulated and analysed as described in Fig. 1. White circles: LPS; Black circles: LPS and GM-CSF. Each bar represents the mean of triplicates +/− SD (C) Nuclear recruitment of NF-κB subunits in FLT3L-derived CD11b^+^ DCs was analyzed by DNA-binding ELISA (p50, p52 and p65) and western blot (c-Rel). Results illustrated were confirmed in two independent experiments.

To clarify whether GM-CSF acts at the level of IL-1β synthesis, of its processing or plays a role in both pathways, DCs and MØ were stimulated overnight with LPS in the absence or in the presence of GM-CSF. Western blot analysis showed five-times higher amounts of the pro IL-1β form in cells stimulated with the combination of LPS and GM-CSF as compared to cells treated with LPS alone (Fig. 3A). Together with IL-1β we measured also the content of the inflammasome components, such as NLRP3, caspase-1 and -11 in untreated and differently stimulated cells. As shown in Fig. 3A, caspase-1 protein levels were similar in all tested conditions in both DCs and MØ, whereas NLRP3 and caspase-11 levels were increased upon stimulation, but without any obvious difference between LPS and LPS/GM-CSF treatments.

In this context, we tested the effect of two different NF-κB inhibitors (Bay11-7082 and Wedelolactone) on DCs treated with LPS alone or the LPS/GM-CSF combination. The secretion of IL-1β was clearly dose-dependently decreased by both inhibitors pointing to a crucial role of GM-CSF in sustaining NF-κB activation (Fig. 3B). The decrease was not caused by a possible cytotoxicity effect of the inhibitors, since a 1–3 µM Bay 11-0782 and 30 µMWedelolactone concentration caused around 80% IL-1β inhibition without any detectable effect on cell viability (Fig. S2). In addition, augmented NF-κB activation via the combination of LPS and GM-CSF could be clearly visualized by measuring a strong augmented nuclear translocation of NF-κB subunits p65, p50, p52 as well as c-Rel only upon stimulation with both stimuli as illustrated in Fig. 3C.

**Figure 4:**
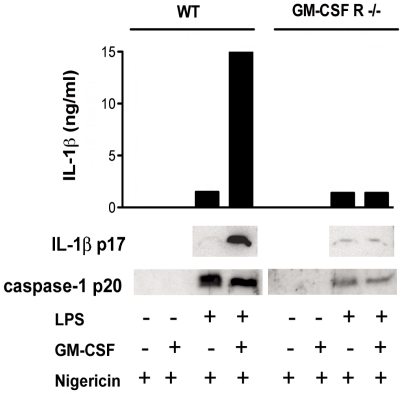
LPS and LPS/GM-CSF induce equal cleavage of caspase-1 in presence of a danger signal. Measurement of bioactive secreted IL-1β p17 and active caspase p20 in serum free culture supernatants of WT and GM-CSFR−/− L929-derived BM MØ treated overnight with 100 ng/ml LPS, 5 ng/ml GM-CSF or a combination of both and then pulsed for 1 h with Nigericin. Upper panel shows the quantification of the amount of IL-1β released measured by ELISA. Results are representative of two independent experiments.

We then analysed the proteolytic processing of pro-caspase-1 triggered by nigericin in WT and in GM-CSF R−/− MØ upon LPS stimulation in presence or absence of GM-CSF. The cleavage of the active form of the enzyme, p20 was clearly detectable in both WT and GM-CSF R−/− cells independently whether GM-CSF was co-applied (Fig. 4). However, the mature IL-1β (p17) was efficiently released in the culture supernatant by MØ stimulated with the LPS/GM-CSF combination, result which could be confirmed either by western blot or by ELISA measurement. These results demonstrate that GM-CSF is crucial for efficient priming of the pro-IL-1β form synthesis via strengthening the NF-κB signalling but it is dispensable for the caspase-1 activation.

### GM-CSFR−/− mice are more resistant to LPS-induced septic shock

**Figure 5:**
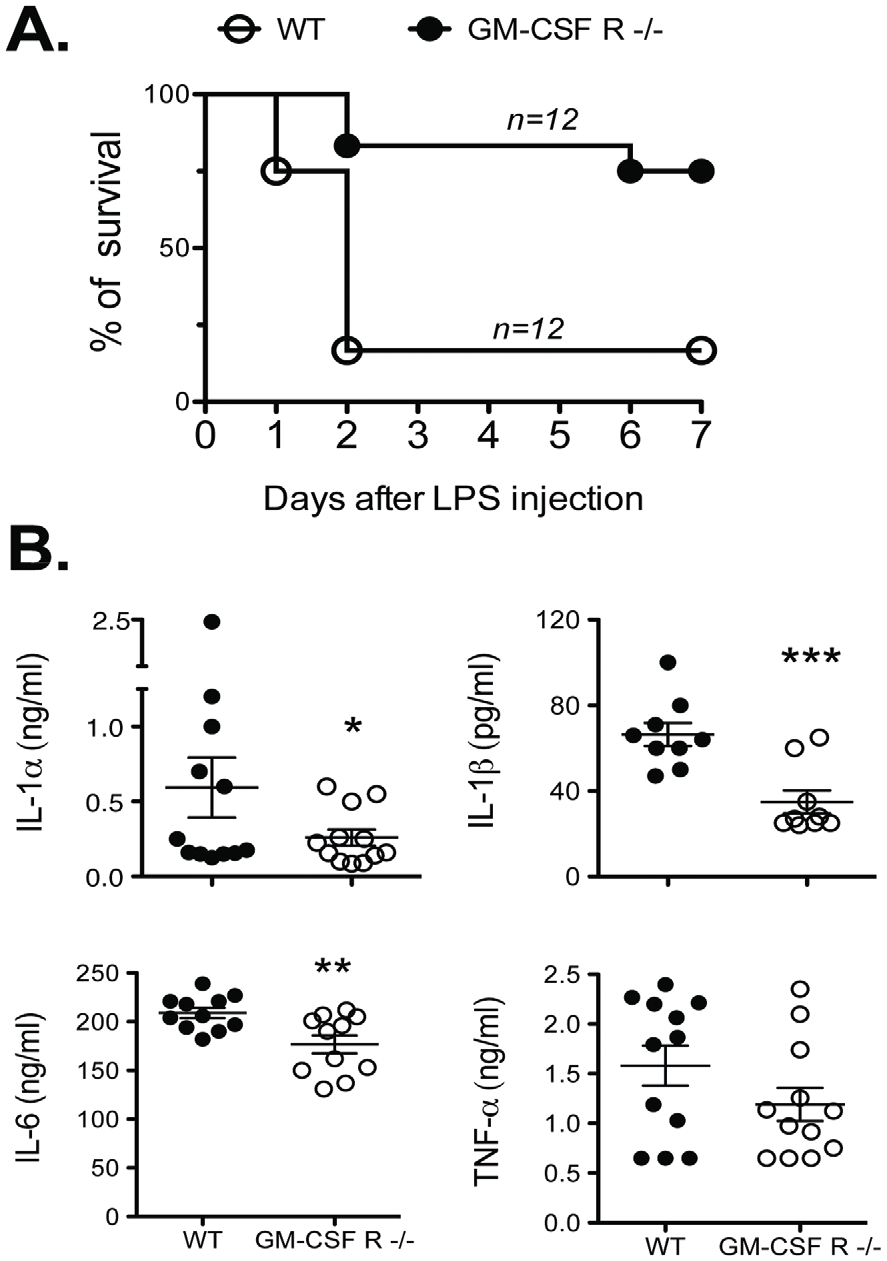
GM-CSF R−/− mice survive LPS-induced septic shock. (A) Survival of WT and GM-CSFR−/− mice ( n = 12 each group) injected i.p. with 50 µg/g body weight LPS. (B) WT and GM-CSFR−/− mice were bled from the retro-orbital plexus 3 h after LPS treatment. Pro-inflammatory cytokines such as IL-1α, IL-1β, IL-6 and TNF-α were measured in the serum by ELISA. The data represent the mean +/− SD of three pooled independent experiments. *<0.1, ** P<0.01, *** P<0.001 (Student's *t* test).

It is commonly accepted, that excessive production of pro-inflammatory cytokines such as TNF-α and in particular IL-1, is one of the major causes of septic shock induced by endotoxin. To determine whether GM-CSF contributes to septic shock, we injected 50 µg/g LPS i.p. and both mouse survival and serum cytokine levels were monitored in WT and GM-CSF R−/− mice. At this high concentration, LPS induces a caspase-1 dependent endotoxin shock caused by elevated levels of pro-inflammatory cytokines like TNF-α and IL-1β [14] [15]. In line with the observed effects of GM-CSF in boosting IL-1 secretion, it is not a surprise that GM-CSF R−/− mice are more resistant to LPS-mediated septic shock. In fact, as shown in Fig. 5A, 80% of the WT mice died within the first two days, whereas 10 out of 12 GM-CSFR−/− mice were resistant to the lethal LPS injection and survived up to 7 days. In addition, GM-CSFR−/− mice had lower levels of serum IL-1β (*** P<0.001), TNF-α (** P<0.01) and IL-1α (* P<0.1) after LPS injection (Fig. 5B).

### Rab39a expression is greatly enhanced by LPS and GM-CSF co-stimulation

**Figure 6:**
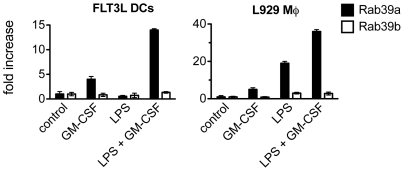
Rab39a mRNA but not Rab39b mRNA is enhanced upon LPS and GM-CSF co-stimulation. FLT3L derived CD11b^+^ DCs and L929-derived BM MØ were stimulated for 24 hours with 100 ng/ml LPS in presence or absence of 5 ng Rab39a and Rab39b expression was calculated using a comparative method for relative quantitation upon normalization to HPRT gene expression/ml GM-CSF. mRNA levels of Rab39a (black bars) and Rab39b (white bars) were determined by qPCR. Rab39a and Rab39b expression was calculated using a comparative method for relative quantitation upon normalization to HPRT gene expression and represented as fold increase of triplicates +/− SD.

It was recently shown that over expression of Rab39a, a member of the RabGTPase family, leads to an enhancement of IL-1β secretion and that its expression is regulated by TLR agonists, like MALP2, LPS and Pam3Cys [16]. To investigate the effect of GM-CSF on its expression levels, we analysed by qPCR the Rab39a and, as a control, the related Rab39b mRNA levels upon DCs and MØ stimulation with LPS and LPS/GM-CSF, respectively. As shown in Fig. 6, in both cell types the combination of LPS and GM-CSF lead to enhanced Rab39a upregulation when compared to LPS and GM-CSF alone. The profile of the Rab39b mRNA, another member of the RabGTPase family but without caspase-1 binding property, remained unchanged.

## Discussion

Although GM-CSF was originally characterized as a haematopoietic growth factor responsible for the differentiation of BM progenitor cells and mobilization of myeloid cells, in the last years GM-CSF has been recognized as a key pro-inflammatory cytokine during inflammation or in response to infection [9] [10], [11]. This property is exploited in many vaccination strategies where GM-CSF is included to boost the immune responses [17] [18] [19] [20] [21]. Similarly to CD40-mediated DC maturation [22], GM-CSF supports the formation of potent “effector” DCs capable in secreting a variety of pro-inflammatory cytokines only when combined with microbial stimuli [23]. In fact, combinations of GM-CSF with different TLR agonists, like LPS, CpG, PolyI∶C and Zymosan augmented clearly IL-12p70 secretion [23] as well as IL-6 and some TNF-α release as described here in this paper. Indeed, GM-CSF was previously shown to regulate cytokine production by MØ due to upregulation of CD14 and TLR4 [24] [25].

Here we describe a novel and unexpected finding that GM-CSF can act as a strong synergistic enhancer of the inflammasome-dependent IL-1β secretion in response to NF-κB activating agonists, such as LPS, TNF-α and Dectin-1 ligands. In fact, unlike TNF-α, IL-6 and IL-12 whose secretion can be maximally increased only about 2-fold by the LPS/GM-CSF co-stimulation, microbial-induced IL-1β secretion can be synergistically augmented by GM-CSF up to 10-fold. Our data strongly suggest that GM-CSF acts as enhancer of the synthesis of the pro IL-1β form. The enhanced pro-IL-1β levels could be explained by the GM-CSF ability to regulate the IL-1β gene expression at transcriptional as well as post-translational levels as shown by Fernandez et al in human polymorphonuclear leukocytes [26].

In addition, we could rule out its role in the cleavage process required for the formation of the IL-1β active form which is actually stimualted by many so called “danger signals” such as extracellular ATP, Alum as well as uric acid. Interestingly, GM-CSF revealed to be the most potent modulator of the IL-1β secretion when compared to other pro-inflammatory cytokines, such as TNF-α, IL-6, INF-γ and M-CSF, which hardly primed its release. Furthermore, the observed synergistic enhancement of pro-IL-1β synthesis upon co-stimulation with LPS/GM-CSF is not a consequence of an augmented GM-CSF-mediated cell survival since no difference in number of viable cells between the two stimulated groups (LPS versus LPS/GM-CSF) was observed during the short window of stimulation (3 h to max 16 h) (unpublished data).

Although we have related the “GM-CSF phenomenon” to increased synthesis of pro-IL-1β it is possible, maybe likely, that GM-CSF signalling could also influence molecular pathways controlling the still mysterious and unconventional secretion of IL-1β. In fact, it is reported that GM-CSF triggers granule exocytosis in human neutrophils [27]. In this context, we show that GM-CSF greatly augmented the expression of the trafficking adaptor Rab39a, a recently discovered GTPase which links caspase-1 to IL-1β secretion. [16]. This suggests that GM-CSF can have a role also in extracellular IL-1β release.

GM-CSF/LPS combination also increased greatly the expression of NLRP3, caspase-1 and caspase-11 mRNAs in several experiments (although not detected in our conditions at protein level, unpublished observations) suggesting that in some unknown situations the protein levels of the most inflammasome components can be further boosted.

How GM-CSF amplifies this massive pro-IL-1β synthesis is still elusive. Based on our results, it is clear that the GM-CSF and LPS induced intracellular pathways have to cross-talk upon co-engagement of the correspondent receptors. It is well documented that GM-CSF activates the Jak2/STAT5, Ras/Raf/MAPK as well as PI3K/Akt pathways through its heterodimeric receptor composed by a major binding unit GMRα and a major signalling unit GMRβ_c_ (reviewed in [10], [28], [29]). Interestingly, there is also evidence suggesting the involvement of GM-CSF in the activation of the NF-κB signalling pathway. Ebner et al. elegantly showed using a two-hybrid yeast system that IκB kinase (IKK) β associates with the GM-Rα subunit [30], whereas Nakamura et al. demonstrated that signals downstream the β_c_ induce a STAT5-dependent increase of NF-κB binding and *trans* activation in murine proB cells [31]. More recently, Meads et al. proposed a new model which suggests TNF receptor-associated factor 6 binding domain (TRAF6) as an intracellular adaptor for GM-CSF-induced NF-κB activation demonstrating for the first time that TRAFs are important signalling intermediates not only for TNFRs and TLRs but also for class I cytokine receptors, such as GM-CSF R [32]. In addition, it has been recently shown that Iκ-Bβ operates as an important co-activator for LPS-induced IL-1β transcription through its recruitment to their specific promoter in complex with two other NF-κB subunits p65/RelA and c-Rel [33], [34]. Our results show that the nuclear translocation of both p65/RelA as well c-Rel was detectable in the case of LPS stimulation but was clearly strongly enforced when DCs were stimulated with the combination of both stimuli.

It is commonly accepted, that excessive production of pro-inflammatory cytokines such as TNF-α and IL-1 is one of the major causes of septic shock induced by bacterial endotoxin. In line with the observed effects of GM-CSF in boosting IL-1 secretion, it is not a surprise that GM-CSF R−/− mice are more resistant to LPS-mediated septic shock. Interestingly, IL-1β deficient mice are sensitive to LPS induced shock while GM-CSF R−/− mice show resistance comparable to caspase-1 [15], [35], caspase-11 [36], ASC [37], Iκ-Bβ [33], [34] and CIAS1 (cryopyrin) [38] deficient mice. The LPS resistance observed in our study is in accordance with data published some years ago by Basu et al. who reported an enhanced tolerance to LPS in GM-CSF deficient mice [39]. Similarly to our results, after LPS treatment reduced circulating levels of IL-1α and IL-6 were detected whereas TNF-αlevels in the serum were comparable to those in control mice. Furthermore, treatment with anti-GM-CSF neutralizing antibody protects mice against a lethal endotoxin dose underlying the role of GM-CSF as endogenous enhancer of LPS-mediated toxicity [40]. In fact, GM-CSF serum content is elevated after LPS injection although in much lower extent than the level of prototype endotoxin-induced TNF-α. However even tiny amounts of GM-CSF (as low as 100 pg/ml) are effective in priming the maturation of DCs and boosting the LPS induced IL-1 production (data not shown).

In conclusion, we have shown here that GM-CSF is a sensitive and strong amplifier of IL-1 release after bacterial infections or after many inflammatory triggers. Since many inflammatory diseases respond to specific IL-1β or caspase-1 blocking therapies [7], our data suggest to include GM-CSF as an additional potential target in the development of more efficient therapeutic strategies against inflammasome-related autoinflammatory diseases. Furthermore, a better understanding of the molecular basis of this synergistic activity mediated by GM-CSF will provide new exciting insights on how to tailor more efficient vaccination strategies.

## Materials and Methods

### Mice

B6.129S1-*Csf2rb^tm1Cgb^*/J (GM-CSF receptor−/−) and C57BL/6 mice were obtained from The Jackson Laboratories (Maine, USA), bred and maintained in the animal facility of the Nanyang Technological University under specific pathogen-free conditions.

### Reagents

TLRgrade™ LPS was purchased from Alexis (Enzo Life Sciences, Alexis, Lausen, Switzerland), Bay 11-7082, Wedelolactone, Alum, Pam3CysSK4 and Curdlan from Sigma (St Louis, MO, USA), MSU, ATP, Nigericin from InvivoGen (San Diego, CA, USA). Following antibodies were used for western blotting: anti-tubulin, anti-caspase-1 p20, anti-c-Rel and anti-USF-2 (Santa Cruz Biotechnlogy, Santa Cruz, CA, USA), anti-IL-1β antibody (R&D), anti-NLRP3 (Alexis), anti-caspase-11 (Biolegend, San Diego, CA, USA). Recombinant GM-CSF, TNF-α, IL-6 and IFN-γ were obtained from Biolegend, M-CSF from Milteny Biotech (BergischGladbach, Germany).

### BM-derived DCs and MØ

GM-CSF and FLT3L-derived BM DCs were generated by incubating freshly prepared BM cells for 8 days in IMDM medium supplemented with 20 ng/ml GM-CSF or 100 ng/ml FLT3L, respectively. In the case of the FLT3L generated DCs, CD11b^+^ cells were purified by magnetic separation (MiltenyiBiotec). Two types of MØ were generated from BM cells using L929 cellconditioned medium (30%) (L929-derived BM Mφ) or 20 ng/ml M-CSF (M-CSF-derived BM Mφ), respectively.

### Cytokine measurements

Cells were stimulated overnight with LPS with or without GM-CSF and subsequently treated for 1 h or 6 h with different danger signals (5 mM ATP, 1 µM nigericin, 100 µg/ml MSU and 200 µg/ml Alum) and cell supernatant was analysed for IL-6, TNF-α, IL-1α and -β by ELISA following manufactures instructions (Biolegend).

### Quantitative RT-PCR

CD11b^+^ fraction of FLT3L generated BM DCs and L929-derived BM MØ were stimulated for 24 hours with 100 ng/ml LPS in presence or absence of 5 ng/ml GM-CSF. Total RNA was extracted using the Pure Link RNA Isolation Kit (Invitrogen) as per manufacturer's instructions and cDNA was prepared. SYBR green I-based quantitative real-time PCR (QPCR) was performed following manufacture's protocol (KabaSyBR FAST qPCR Kit, Kababiosystem, Woburn, MA). Rab39a and Rab39b expression was calculated using a comparative method for relative quantitation upon normalization to HPRT gene expression.

### Western blotting

For detection of the pro IL-1β, caspase-1, caspase-11, NLRP3 and tubulin, as an internal control, lysates of L929-derived MØ and FLT3L-derived DCs were resolved by SDS-PAGE and equal amounts transferred onto nitrocellulose membranes. The blots were developed by chemiluminescence according the manufactures instructions (Perkin Elmer, Waltham, MA, USA).

For detection of the caspase-1 cleaved p20 subunit as well as of the bioactive IL-1β p17 form, L929-derived BM MØ were incubated overnight untreated or with 5 ng/ml GM-CSF, 100 ng/ml LPS or the combination of 5 ng/ml GM-CSF and 100 ng/ml LPS, respectively. After 1 h stimulation with 1 µM nigericin, supernatants were collected and equal amounts of concentrated fractions were separated by SDS-PAGE.

To measure nuclear NF-κB subunits p65, p50, p52 and c-Rel levels, FLT3L-derived CD11b^+^ DCs were kept untreated or stimulated for 20 h with 5 ng/ml GM-CSF, 100 ng/ml LPS or the combination of 5 ng/ml GM-CSF and 100 ng/ml LPS, respectively. NF-κB DNA binding activity of p65, p50, p52 was determined using a DNA-binding ELISA kit (TransAM NF-κB transcription factor kit, Active Motif, Carlsbad, CA, USA) using equal amounts of nuclear extracts. c-Rel and USF-2 as an internal standard were monitored by western blotting.

### LPS-induced septic shock

WT and GM-CSFR−/− mice (6–8 weeks old) were i.p. injected with 50 µg/g of LPS from E.coli (055:B5, Sigma) The mice were monitored for sign of septic shock and for lethality over a period of 7 days. For serum cytokine analysis, mice were bled 3 h after treatment.

### Data analysis

Calculations, statistical analysis and graphs were performed on Graphpad Prism 4.0 (Graphpad Software, San Diego, USA).

### Ethics statement

This study was carried out in strict accordance with the recommendations of the NACLAR (National Advisory Committee for Laboratory Animal Research) guidelines under the Animal & Birds (Care and Use of Animals for Scientific Purposes) Rules of Singapore. The protocol was approved by the by the Institutional Animal Care and Use Committee (IACUC) of the Nanyang Technological University of Singapore (Approval number: ARFSBS/NIE A0135). All efforts were made to minimize the suffering.

## Supporting Information

Figure S1 **CD11b^+^ FLT3L generated DCs (3×10^5^/well) were primed for 24 h with 100 ng/ml LPS in absence (white bars) or presence (back bars) of 5 ng/ml GM-CSF, 50 ng/ml M-CSF, 50 ng/ml TNF-α, 50 ng/ml IL-6, 50 ng/ml IFN-γ.** 5 mM ATP was added for the last 1 h as a danger signal. Released IL-1β was measured in the culture supernatants by standard ELISA and each value represents the mean of triplicates +/− SD.
(TIFF)

Figure S2 **Effect of Bay 11-7082 and Wedelolactone on DC viability.** FLT3L derived DCs were pre-treated for 30 min with a different concentration range of inhibitors, stimulated as described in Fig. 1. White bars: LPS; Black bars: LPS and GM-CSF Cells were stained with propidium iodide and analyzed by FACS gating of FCS/SSC. % of viable cells is indicated.
(TIF)
