# Supplementary figures and images for: GM-CSF Signalling Boosts Dramatically IL-1Production
Source: PLoS One. 2011 Jul 28;6(7):e23025. doi: 10.1371/journal.pone.0023025 (PMC3145786; doi:10.1371/journal.pone.0023025)

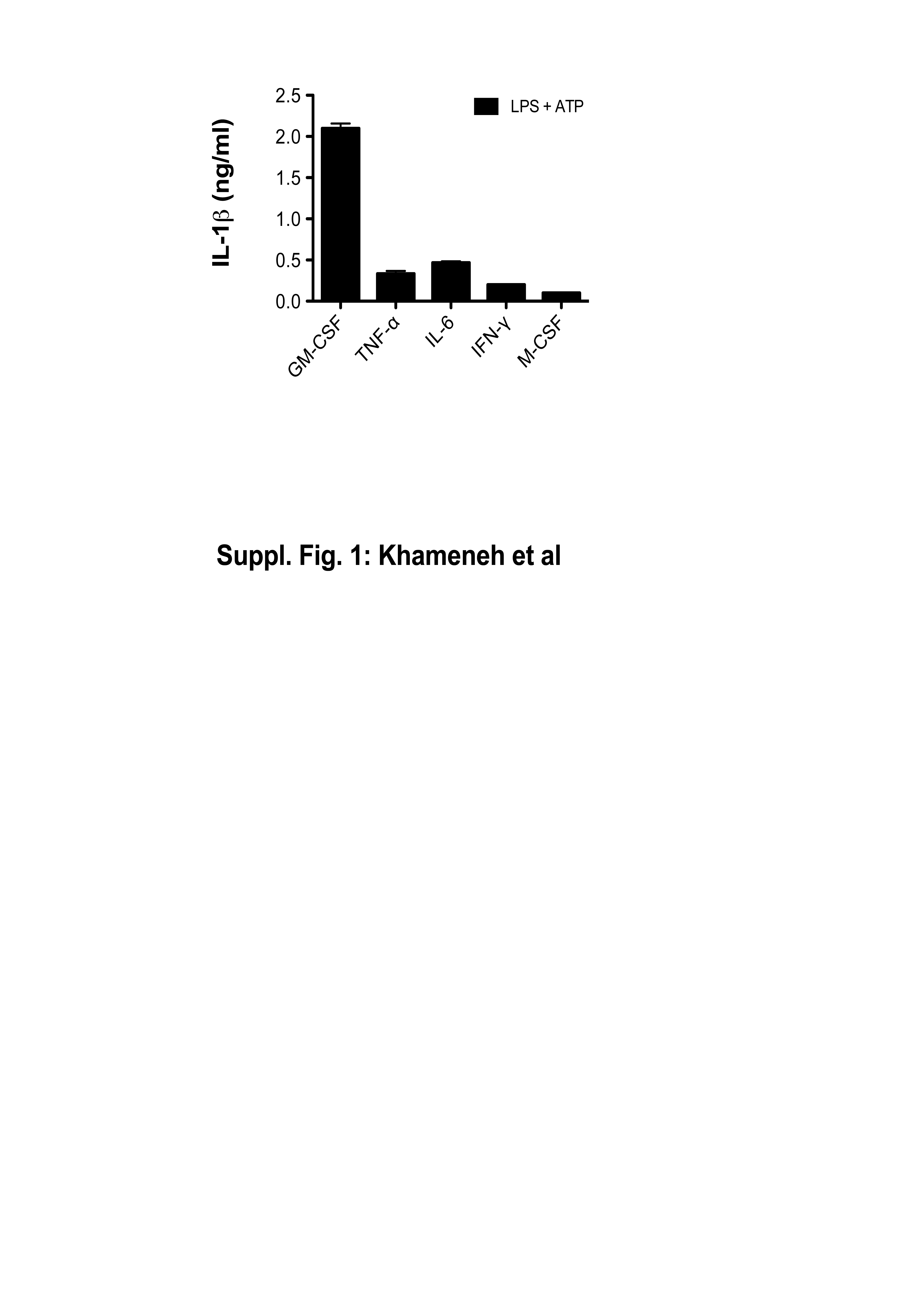

Supplement: Figure S1 — CD11b+ FLT3L generated DCs (3×10^5^/well) were primed for 24 h with 100 ng/ml LPS in absence (white bars) or presence (back bars) of 5 ng/ml GM-CSF, 50 ng/ml M-CSF, 50 ng/ml TNF-α, 50 ng/ml IL-6, 50 ng/ml IFN-γ. 5 mM ATP was added for the last 1 h as a danger signal. Released IL-1β was measured in the culture supernatants by standard ELISA and each value represents the mean of triplicates +/− SD. (TIFF) [file pone.0023025.s001.tiff]

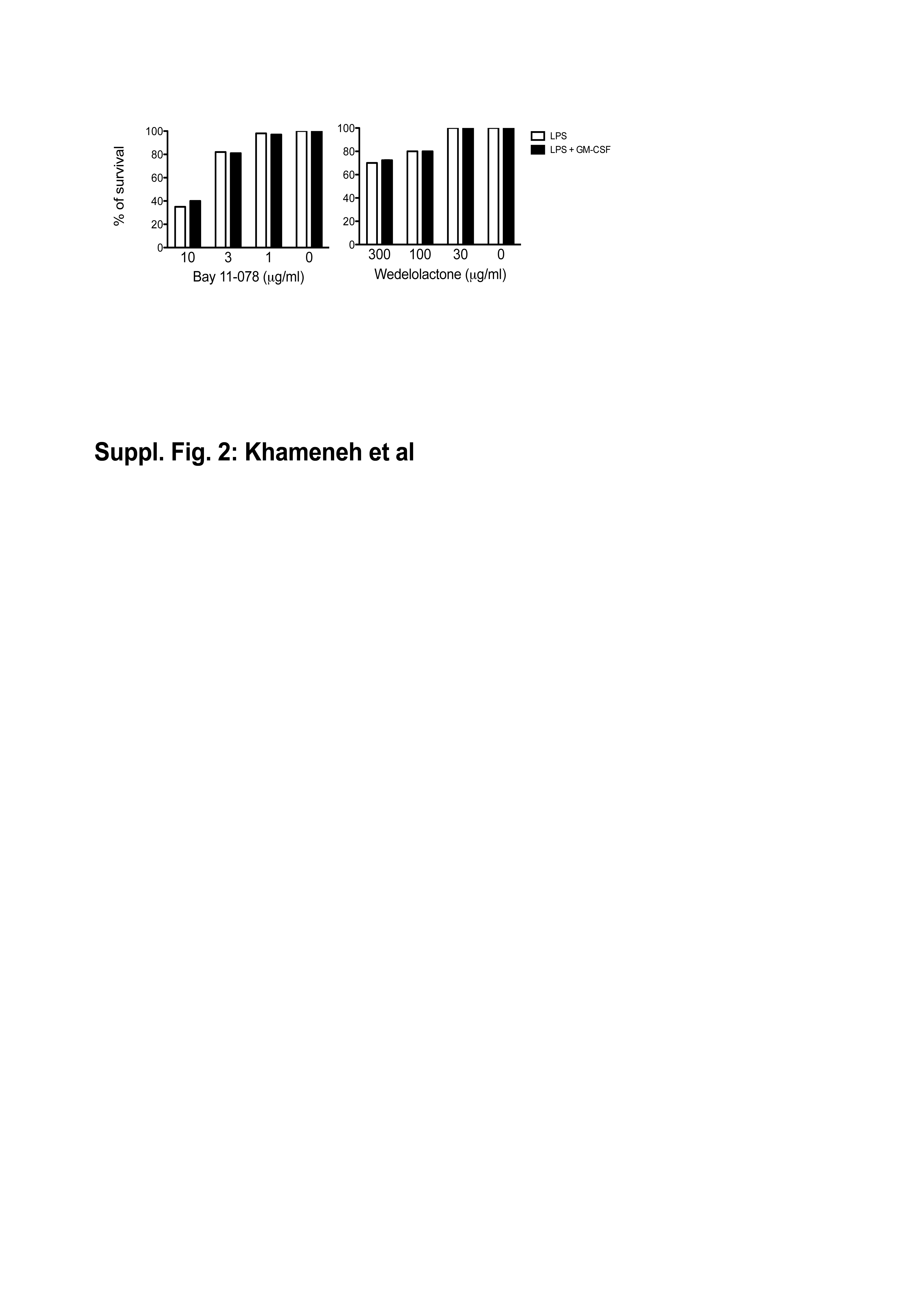

Supplement: Figure S2 — Effect of Bay 11-7082 and Wedelolactone on DC viability. FLT3L derived DCs were pre-treated for 30 min with a different concentration range of inhibitors, stimulated as described in Fig. 1. White bars: LPS; Black bars: LPS and GM-CSF Cells were stained with propidium iodide and analyzed by FACS gating of FCS/SSC. % of viable cells is indicated. (TIF) [file pone.0023025.s002.tif]
